# The unseen architects of metastasis: coagulation factors in pre-metastatic niche development

**DOI:** 10.1186/s12964-026-02727-0

**Published:** 2026-02-11

**Authors:** Haoyu Huang, Xiangtong Lu, Yang Liu, Yuxin Zhang, Jianhao Zhan, Wenjuan Zeng, Chengpeng Sun, Benjie Li, Yujun Zhang, Qixian Wang, Zijun Ding, Lingling Yang

**Affiliations:** 1 Department of Gastroenterology, The Second Affiliated Hospital, Jiangxi Medical College, Nanchang University, No. 1 Minde Rd., Nanchang, Jiangxi 330006 China; 2 Department of Pathology, The Second Affiliated Hospital, Jiangxi Medical College, Nanchang University, No. 1 Minde Rd., Nanchang, Jiangxi 330006 China; 3 Jiangxi Medical College, Huankui Academy, Nanchang University, Nanchang, 330006 Jiangxi China; 4 Queen Mary School, Jiangxi Medical College, Nanchang University, Nanchang, 330006 Jiangxi China; 5 School of Ophthalmology and Optometry, Jiangxi Medical College, Nanchang University, Nanchang, 330006 Jiangxi China

**Keywords:** Pre-metastatic niche, Coagulation factors, Tumor metastasis, Platelets, Fibrinogen, Thrombin

## Abstract

Cancer metastasis, the leading cause of cancer-related deaths, is a complex process driven by the interplay of multiple factors. Pre-metastatic niche (PMN), formed in distant organs before the arrival of circulating tumor cells (CTCs), provides a favorable environment for CTC colonization and growth. While traditionally known for their role in hemostasis, coagulation factors are increasingly recognized for their significant contributions to tumor development and progression. This review first discusses the multifaceted role of coagulation factors in preparing the PMN for tumor cell colonization. We explore the mechanisms by which coagulation factors, including platelets, fibrinogen, thrombin, and tissue factors (TFs), contribute to PMN formation and metastasis. These factors, through their interactions with tumor cells and the surrounding microenvironment, activate endothelial cells, recruit immune cells, release pro-angiogenic factors, and promote inflammation and extracellular matrix (ECM) remodeling, ultimately facilitating tumor cell colonization and growth. Understanding the interplay between coagulation and metastasis helps provide novel insights and directions for clinical anti-cancer treatment.

## Introduction

Cancer metastasis is the primary cause of cancer-related mortality, driving extensive research into its underlying mechanisms to identify preventive strategies [1]. Metastasis, a complex process involving multiple molecular pathways, relies on the entry and survival of (CTCs) in target organs—yet CTCs are highly vulnerable outside the tumor microenvironment (TME) [2]. This underscores the critical role of the target organ’s local microenvironment, a relationship first outlined by Stephen Paget’s 1889 “seed and soil” hypothesis [3]. Prior to CTC (“seed”) arrival, target organs remodel to form a PMN (“soil”) that supports tumor cell survival [4]. Growing research has shed light on the mechanisms of PMN formation, offering new insights into understanding cancer metastasis. As we previously reviewed, various cellular and molecular components, such as tumor-derived soluble factors (TDSFs), coagulation factors, bone marrow-derived cells (BMDCs), immune cells, and extracellular vesicles all promoted the development of PMN [5, 6].

Traditionally regarded for their roles in hemostasis and thrombosis, coagulation factors have been increasingly shown to play critical roles in tumor development and progression over the past few decades [7], regulating multiple key biological behaviors of tumors. As early as 1986, Dr. Harold Dvorak first demonstrated that the coagulation factors function beyond wound healing to promoting tumor growth and proliferation [8]. and subsequent studies have further established a robust link between these factors and cancer metastasis [9–11]. Key coagulation components, including platelets, thrombin, and fibrinogen, interact directly with tumor cells to drive metastasis in cancers such as lung, breast, and colorectal cancer (CRC): platelets suppress T cell activity via PD-L1 to enable immune evasion and use P-selectin to form protective complexes with tumor antigens that enhance vascular adhesion; thrombin activates PAR1/PAR4 on tumor cells to induce cytoskeletal changes and upregulate matrix metalloproteinase 9 (MMP-9), while also promoting ECM degradation and angiogenesis through vascular endothelial growth factor (VEGF) and Basic fibroblast growth factor secretion; fibrinogen, once converted to fibrin by thrombin, protects CTCs from apoptosis and NK cell clearance, and drives invasion via PI3K/Akt and focal adhesion kinase (FAK) pathway activation [5, 12, 13].

Our recent work adds to this body of knowledge: a new review reveals that fibrinogen primarily enhances PMN formation by inducing immune suppression, inflammation, ECM remodeling, and angiogenesis [5], while our in vivo experiments showed that FGG downregulates VE-cadherin in CRC tissues and upregulates CD31 in liver tissues, ultimately increasing the number of metastatic liver nodules in mouse models [14]. Despite these advances, the specific role of procoagulant processes in PMN formation during cancer metastasis remains poorly understood. In this review, we characterize the PMN formation and first discuss how diverse coagulation components promote metastasis by shaping the PMN, providing novel insights and clinical directions for targeting coagulation factors to inhibit cancer metastasis.

## A brief overview of PMN

### Spatio-temporal order of PMN shaping

**Fig. 1:**
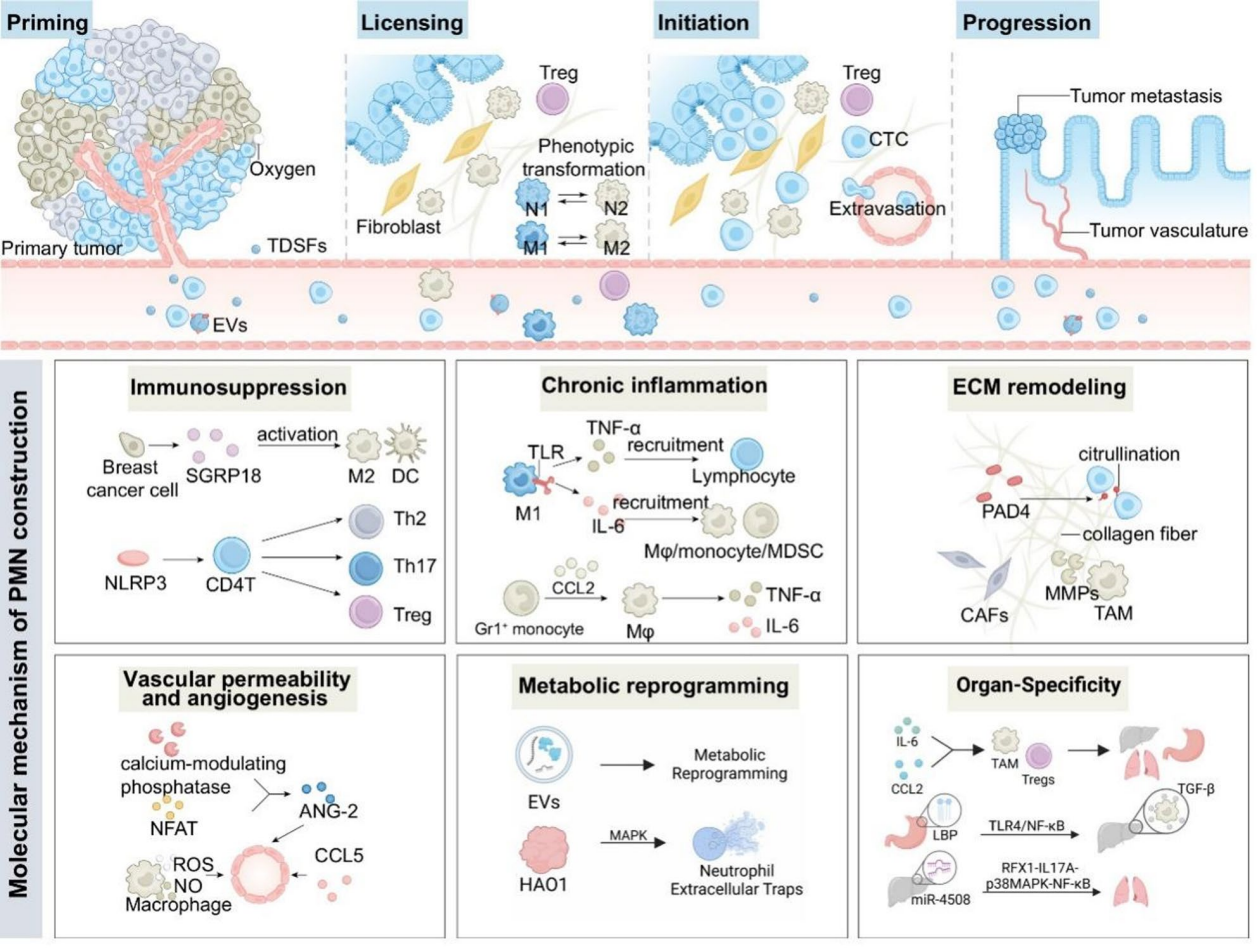
Spatial and temporal sequence and molecular mechanism of PMN formation. The spatiotemporal sequence of PMN formation is divided into four stages, during which primary tumors shape PMN by reprogramming the stromal microenvironment of potential metastatic organs through TDSFs and BMDCs. Six major molecular mechanisms drive this process. First, TDSFs promote immunosuppression and chronic inflammation by recruiting immunosuppressive cells and triggering inflammatory factor release. Second, ECM remodeling facilitates tumor metastasis by modulating angiogenesis and enabling tumor cells to evade immune surveillance. Third, angiogenesis and metabolic reprogramming improve the stromal microenvironment, providing CTCs with oxygen and nutrients to support their colonization, growth, and proliferation. Collectively, tumor cells secrete diverse biomolecules that orchestrate PMN formation in target organs through multiple signaling pathways, thereby establishing the organ-specific basis for metastasis

PMN formation is a prerequisite for tumor metastasis. As core components shaping the PMN, TDSFs, BMDCs, and stromal cells of secondary organs and their derivatives interact with one another in a defined spatio-temporal sequence [4]. This ordered process of PMN assembly has been divided into four stages by researchers (Fig. 1).

The first stage is initiation, during which hypoxia is a critical challenge in primary tumor growth. Stimulated by the hypoxic microenvironment, primary tumors secrete various soluble factors, including TDSFs, exosomes, and other molecular components. We have reviewed that TDSFs mainly include chemokines (e.g., CXCL1, CCL2, CCL9, and CCL15), and cytokines (e.g., VEGF-A, PGF, TNF-α, TGF-β, SDF-1, G-CSF, S100A8, S100A9, and TIMP-1), which act in multiple ways to induce PMN formation through recruiting BMDCs or mediating ECM remodeling [6]. These soluble factors regulate PMN formation in distant metastatic organs or in non-primary tumor areas within the same organ. However, the PMN at this time cannot support CTCs colonization and proliferation.

The second stage is recruitment, during which BMDCs, myeloid-derived suppressor cells (MDSCs), and regulatory T cells (Tregs) are recruited to potential metastatic organs under specific TDSFs, such as CCL2 and CXCL1. These TDSFs exert chemotactic effects by binding to corresponding receptors on target cells, facilitating the subsequent maturation of the PMN. The CCR2 + tumor-associated macrophages (TAMs) were mobilized to the liver by CRC-derived CCL2 to promote an immunosuppressive PMN to facilitate CRC liver metastasis in mice models and human samples [15]. CXCR2 + neutrophils homing to the liver by the CXCL1-CXCR2 signaling axis played a crucial role in initiating PMN via upregulating inflammatory cytokines, such as S100A8 and S100A9 [16].

The third stage is colonization, during which PMN matures following “education” by specific TDSFs (e.g., TGF-β, IL-6, CCL5, TNF-α) and BMDCs from the primary tumor [17]. A PMN characterized by immunosuppression, inflammation, angiogenesis, or a reprogrammed ECM not only reactivates dormant disseminated tumor cells but also facilitates their colonization at the target organ. The core of this stage lies in the successful extravasation, survival, and initial adaptation of CTCs at the distant site before formation of any macroscopic lesion. Successful colonization depends on intricate crosstalk between CTCs and the PMN, such as CTC adhesion to vascular endothelium via specific adhesion molecules (e.g., intercellular adhesion molecule-1 (ICAM-1), VCAM-1) [18].

The fourth stage, metastatic outgrowth, is critical as it generates clinically detectable lesions. Tumor cell clones that have colonized the target site initiate proliferative expansion, driving the development of overt metastases [19]. This outgrowth is fueled by processes such as angiogenesis to secure nutrient supply, and further adaptation of the tumor cells to the local microenvironment. Concurrently, two distinct events shape the metastatic phenotype: continuous seeding of additional CTCs into this permissive niche increases the quantity of metastatic lesions, while evolutionary pressures within the new microenvironment select for subclones with additional genetic or epigenetic alterations—leading to a qualitative leap in malignancy (enhanced aggressiveness). Thus, this stage culminates in the establishment of mature, destructive metastatic lesions.

### Molecular mechanisms of PMN shaping

Cao et al.. first proposed six major PMN features (immunosuppression, inflammation, and others) determining CTCs’ fate [4]. PMN shaping is multi-component and multi-stage. Briefly, TDSFs(e.g., VEGF, TGF-β, CCL2, IL-6) and BMDCs from primary tumors are modified or regulated to adapt to metastatic organs, thereby creating suitable microenvironments for CTCs based on organ properties. Thus, these microenvironments constitute the PMN (Fig. 1).

#### Immunosuppression

Immunosuppression in the TME drives cancer progression by allowing tumor cells to evade immune detection, resist therapy, and proliferate unchecked [20]. Immune cells (e.g., Tregs, MDSCs, macrophages) accumulate in or are recruited to the PMN, forming an immunosuppressive milieu that supports metastatic cell survival and colonization [21]. Alveolar macrophages promote lung metastasis by recruiting MDSCs via CXCL10/CXCR3 and TLR4/CCL12 interactions [22]. Breast cancer-secreted GRP78 inhibits DC function, induces M2 macrophage polarization, establishes a hepatic immunosuppressive PMN, and enhances CTC colonization [23]. In PDA macrophages, NLRP3 signaling activation boosts immunosuppressive macrophage proliferation and drives CD4 + T cell differentiation into Th2, Th17, and Tregs, fostering immune tolerance [24]. Forming an immunosuppressive PMN requires coordinated immune cell recruitment and functional modification (e.g., impaired pathogen recognition/attack, immunosuppressive factor production), critical for CTC colonization, proliferation in distant organs, and metastasis.

#### Chronic inflammation

Chronic inflammation is a key driver of tumor progression, supporting immune evasion, tissue remodeling, angiogenesis, and pro-tumor immunity [25]. Tumor cells secrete pro-inflammatory cytokines/chemokines to shape the PMN [26]. A decade ago Qian et al.. reported breast cancer-derived CCL2 recruits Gr1^+^ inflammatory monocytes to lung PMN [27]. Tumor-derived cytokines (e.g., IL-6, IL-8, TNF-α) drive inflammation and immune cell recruitment to distant organs during PMN formation [28]. For example, IL-6/STAT3 recruits MDSCs (CD11b^+^/Gr1^+^) and polarized macrophages (CD11b^+^/F480^+^) to pre-metastatic lungs, forming a pro-inflammatory, immune-evasive milieu [29]. Likewise, triple-negative breast cancer-secreted TNF-α/IL-1β recruit immunosuppressive cells, macrophages, lymphocytes, and neutrophils to enhance tumor aggressiveness [30]. Notably, VEGF, TGF-β, and IL-6 [31–33] drive PMN tissue remodeling and angiogenesis, inducing ECM and neovascularization to support tumor cell invasion.

#### Vascular angiogenesis and permeability

Angiogenesis in the TME is fundamental for tumor growth, survival, and metastasis, providing vascular support for primary tumor intravasation and priming distant organs for colonization [34]. PMN high permeability and neoangiogenesis support CTC oxygen/nutrient access, environmental interactions, and metastasis. TME stromal cells (notably fibroblasts) enhance PMN vascular permeability (VP) and angiogenesis. For instance, breast cancer lung metastasis cancer-associated fibroblasts (CAFs) highly express LncSNHG5 to stabilize ZNF281 mRNA and activate lung endothelial P38 MAPK [35]; pancreatic cancer TAMs secrete tenascin C, NO, TNF to promote angiogenesis/permeability for invasion [36]. Moreover, tumor-derived EVs—CRC miR-25-3p [37], breast cancer Cav-1 [38], esophageal cancer Circ-ZNF609 [39], lung cancer miR-3157-3p [40]—drive PMN vascular instability and vascularization.

#### Extracellular matrix remodeling

ECM remodeling drives tumor metastasis by modulating cell signaling, promoting sclerosis, and regulating TME angiogenesis/immune escape [41]. Once PMN is primed via ECM remodeling, ECM acts as a key physical scaffold for metastatic cell invasion [42]. CRC cells secrete peptidylarginine deiminase 4 to citrullinate hepatic type I collagen, enhancing CTC-stromal adhesion and hepatic metastasis [43]. In breast cancer mouse models, ECM remodeling proteins (Col4A1, Col4A5) and degrading enzymes (MMP-2/-3/-4) change significantly prior to lung metastasis [44]. Likewise, melanoma TDSFs activate p38α kinase to upregulate lung fibroblast activation protein, promoting lung fibronectin accumulation and metastasis [45]. TAMs secrete MMPs, TGF-β, IL-10 to promote ECM deposition and PMN establishment [46, 47]. Beyond structural support for tumor cell migration, PMN ECM remodeling shields metastatic cells from T/NK cells, preventing immune recognition and enabling immune evasion [48, 49]. ECM remodeling, a hallmark of PMN formation, acts as both scaffold and active participant to promote metastatic colonization in distant organs.

#### Metabolic reprogramming

Metabolic reprogramming alters cellular metabolism, enabling cells to adapt to the microenvironment and drive progression [50]. This metabolic shift—altering nutrient processing, energy generation, and environmental interactions—critical primes PMN for metastatic colonization [51]. Lung mesenchymal cells suppress lipid metabolism via IL-1β-induced hypoxia, causing neutrophil lipid accumulation that boosts breast cancer lung metastasis during PMN formation [52, 53]. Oxalate accumulation (e.g., HAO1) activates MAPK/NADPH oxidase, promoting neutrophil extracellular traps to form a breast cancer lung PMN [54]. For bone metastasis, tumor cells hijack specific iron-transporting macrophages in the bone marrow niche, diverting iron to fuel metabolic adaptation and PMN colonization. Hypoxia further upregulate globin genes (e.g., HBB) to mimic erythroblasts, enhancing their survival in the bone metastatic niche [55]. Jiang et al. reviewed that tumor-derived EVs drive PMN formation via metabolic reprogramming (glucose, fatty acid, lipid, amino acid metabolism) [54]. These studies show tumor signals induce PMN metabolic changes (glycolysis, lactate production, lipid metabolism) to favor cancer cells. Immune cell glycolysis/lactate metabolic reprogramming promotes macrophage polarization and immuno-suppressive PMN formation [56]. Primary tumors remodel nutrient use and metabolic pathways, supporting key PMN modifications: angiogenesis, immune evasion, and ECM reorganization.

#### Organ-specificity

Organ specificity or organotropism, refers to different cancers preferentially metastasizing to specific organs [57], a selectivity driven by organ microenvironments that support CTCs colonization. PMN development relies on crosstalk between primary tumors, the circulatory system, immune cells, and target organs to sustain CTCs. Primary tumor-secreted cytokines/chemokines (e.g., IL-6 [58], CCL2 [59]) recruit immune cells (macrophages, Tregs) to distant organs, forming an immunosuppressive niche. Yuan et al. linked breast cancer exosomal miR-21 to 80% of osteolytic bone metastases [60], while gastric cancer LPS and hepatocellular carcinoma (HCC) exosomal miR-4508 drive liver and lung PMN formation, respectively [61, 62]. Thus, tumor-secreted biomolecules (exosomes, cytokines) shape organ-specific PMNs, endowing metastasis with organ tropism and making specific tissues more “hospitable” to matched primary tumor metastases.

## Platelets contributing to PMN formation

CTCs and platelets engage in a critical pro-metastatic interaction that drives tumor dissemination via multiple coordinated mechanisms [63, 64]. First, platelet surface P-selectin (CD62P) mediates initial adhesion to CTC receptors, after which fibronectin acts as a molecular bridge to stabilize complexes between platelet integrin αIIbβ3 and CTC integrin α5β1, strengthening their binding [65–67]. Platelets then form microaggregates around CTCs, physically masking immune recognition sites and enabling CTC immune evasion by inhibiting NK cell-mediated killing [68]. Activated platelets secrete growth factors and cytokines that remodel the ECM and induce epithelial-mesenchymal transition (EMT) in CTCs, thereby enhancing CTC invasiveness and transendothelial migration capacity [69]. Finally, platelet-derived factors promote PMN formation, establishing a favorable microenvironment for CTC homing and colonization in target organs. this multifaceted CTC-platelet crosstalk accelerates distant metastasis through the synergistic effects of adhesion reinforcement, immune evasion, EMT induction, and PMN formation [70].

### Tumor cells activate platelets

Platelets are activated by tumor cells via direct physical interactions, signaling molecule release, and TME changes. Tumor-expressed PSGL-1 binds platelet P-selectin to activate platelets [71]. Other tumor-expressed adhesion molecules (integrins αIIbβ3/α2β1 [72], podoplanin [73]) bind platelet receptors to induce granule release. Tumor-secreted bioactive substances (cervical cancer ADP [74], lung cancer IL-6 [75], thromboxane A2 (TXA2), G protein-coupled receptors, prostaglandin E2 [76]) promote platelet aggregation/activation. Tumor exosome/EV-carried IL-8 and CD63 directly activate platelets upon contact [77, 78]. Tumor-activated platelets degranulate, releasing bioactive molecules that drive tumor progression and PMN formation to support metastasis.

Notably, tumor-induced platelet activation enables platelet-CTC-immune cell heteroaggregates, critical for metastasis [79]. These complexes (platelets, CTCs, immune cells like neutrophils/monocytes/NK cells) boost CTC-endothelial adhesion and shield CTCs from circulatory shear stress [80]. The platelet cloak reduces blood flow-induced damage, enhancing CTC survival beyond the primary TME. Preclinical/clinical studies highlight diverse platelet-tumor interactions: canonical ligand-receptor binding (e.g., P-selectin/PSGL-1, integrin αIIbβ3/fibrinogen) [71, 72], platelet-derived paracrine signaling (e.g., PDGF-BB), and EV-mediated crosstalk [71]. Moreover, platelet-tumor adhesion induces CTC phenotypic changes (e.g., adhesion molecules, MMPs) to enhance distant colonization [81]. These interactions reinforce CTC protection, supporting subsequent PMN formation.

### Platelets promote thrombosis in PMN

Tumor cells establish a tripartite molecular network to activate platelets—via direct contact, soluble factors secretion, and exosome targeting—thereby laying the groundwork for subsequent coagulation and PMN formation. For direct interactions, PSGL-1 on tumor cells specifically binds P-selectin on platelets, initiating intracellular signaling cascades that trigger platelet activation; this adhesion pathway is clinically linked to metastatic potential [71]. Tumor Integrins αIIbβ3/α2β1 recognize ligands, inducing intraplatelet granule pre-assembly and priming while strengthening platelet–tumor cell adhesion stability [72]. Tumor podoplanin binds the platelet receptor CLEC-2 and activates the Src–Syk–PLCγ2 axis, directly inducing platelet aggregation and degranulation, a key node for tumor-triggered platelet activation [73].In indirect activation, cervical cancer cell-released ADP engages platelet P2Y12 receptor, amplifying intraplatelet calcium signaling to promote aggregation [74]. Lung cancer cell-released IL-6 acts through the JAK/STAT3 pathway bidirectionally, directly activating platelets while remodeling the TME to enhance platelet responsiveness [75]. Tumor-derived TXA2, PGE2, and related ligands bind platelet GPCRs, synergistically amplify platelet activation by promoting vasoconstriction and modulating the affinity of platelet surface receptors [76].Tumor exosomes/EVs function as targeted activators: surface-associated IL-8 and CD63 directly trigger platelet membrane fusion and signaling upon contact, a proximity-based mode that increases activation efficiency and circumvents fluid milieu dilution [77, 78]. These three mechanisms synergize to rapidly convert platelets from resting to activated state, providing the necessary prerequisite for subsequent pathological processes.

Platelets activated by tumors undergo degranulation to release a large repertoire of bioactive molecules, establishing a core regulatory pathway that links coagulation generation with the formation and activation of PMNs, propelling tumor-associated pathological processes. On the coagulation side, ADP from activated platelets further stimulates P2Y12 receptors on circulating platelets, creating a positive feedback loop for platelet aggregation; synergizing with TXA2, it promotes platelet adhesion to endothelial injury sites and formation of the primary hemostatic plug [74, 76]. Platelet-derived procoagulant factors (e.g., platelet factor 4 (PF4), thromboxane) activate the coagulation cascade, accelerating fibrinogen-to-fibrin conbersion to form a stable thrombotic network that provides a physical shield for tumor immune evasion and alters local hemodynamics to facilitate metastasis. For PMNs recruitment and activation, platelet-derived IL-6 functions as a central proinflammatory cue recruiting bone marrow–derived PMNs to the TME and enhancing their vascular endothelium binding by upregulating neutrophil adhesion molecules [75]. IL-8 released through platelet degranulation, together with tumor exosome-delivered IL-8 signals, jointly activates neutrophil NETosis to induce formation of neutrophil extracellular traps (NETs) [77, 78]. NETs capture tumor cells via their DNA–histone scaffold and intensify coagulation by activating factor XII and inhibiting anticoagulant activities, establishing a positive cycle of “coagulation activation → PMNs recruitment → NET formation → further coagulation.” Notably, tumor–platelet binding complexes via PSGL-1/P-selectin and podoplanin/CLEC-2 serve as PMNs adhesion anchoring points, promoting local PMNs accumulation and activation [71, 73]. Integrin αIIbβ3/α2β1-mediated adhesion stabilizes platelet–tumor cell aggregates, creating a localized microenvironment concentrating coagulation factors and fostering PMNs activation [72]. Key molecules in each cited study play specific roles in this network, constituting the molecular basis of tumor-associated thrombosis and PMNs-mediated pathological injury.

### Platelets promote an immunosuppression PMN

Detached tumor cells are highly susceptible to immune elimination, making pre-establishment of an immunosuppressive PMN in target organs indispensable for their successful colonization. Platelets serve as key drivers of TME immunosuppression by mediating immune evasion [82]. Specifically, activated platelets adhere to tumor cells via integrins and fibrin, forming a three-dimensional coating that acts as a physical barrier. This structural shield blocks immune surveillance by natural killer (NK) cells and cytotoxic T lymphocytes, safeguarding tumor cells from elimination [83, 84], while nanoparticles targeting activated platelets disrupt this tumor-platelet interaction to abrogate the protective effect [85]. Beyond physical shielding, platelets actively crosstalk with immune cells, including MDSCs, NK cells, and T cells, to shape immunosuppressive TME and PMN [86]. Tumor-associated platelets synergize with MDSCs to amplify immunosuppression [87]: they secrete CXCL4 to induce monocyte differentiation into MDSCs [88], release PDGF-BB to enhance MDSC infiltration in pre-metastatic lungs [71], and produce PAF that drives polymorphonuclear MDSC differentiation (mechanism yet to be fully elucidated) [89]. Collectively, these platelet-driven mechanisms encompassing structural and immunomodulatory aspects fortify the immunosuppressive barrier, allowing detached tumor cells to escape immune surveillance and elimination and establish metastatic colonies in distant organs.

Platelets play multifaceted roles in tumor immunity and metastasis by interacting with T/NK cells, mediating immunosuppression and PMNs formation. Platelet surface PD-L1 binds to PD-1 on T cells, forming a protective shield for tumor cells to evade immune surveillance, as demonstrated in PD-L1-negative tumors where platelet-derived PD-L1 suppresses anti-cancer immune cell activity [90, 91]. Platelets facilitate tumor immune escape and metastasis through crosstalk with the PD-1/PD-L1 pathway and FOXP3 + Tregs, reinforcing immunosuppressive TME [92]. In HCC, however, platelets exert anti-tumor effects by releasing CD40L via the P2Y12 receptor, activating CD8 + T cells and inhibiting tumor growth [93]. Platelet activation impairs the degranulation, perforin secretion, and target cell lysis of CD4^+^/CD8^+^ T cells, promoting tumor invasion and metastasis [94]. Platelets drive lung metastasis independently of NK cells, with temporal discrepancies between platelet-mediated pro-metastatic effects and NK cell-mediated anti-metastatic functions in B16F1 melanoma. Intriguingly, unlike lung metastases, B16F1 liver metastases increases without platelets, indicating a platelet-independent mechanism for hepatic metastatic progression [83]. Other factors, including PF4 and MMP, also promote immune cells the recruitment to the PMN to suppress local immune responses. Tranditionally, PF4 is primarily stored in platelets and synthesized by megakaryocytes [95]. However, within the specific pathological context of the pre-metastatic microenvironment, activated myeloid cells, including the Ly6G^+^CD11b^+^ neutrophil sub-population, have emerged as an important source of PF4 secretion. The diversity of PF4-producing cell types and dynamic changes in their expression levels during tumor progression may underlie the unique functional roles of this factor. Additionally, platelets facilitate androgen receptor-negative cell invasion by upregulating the MMPs (MMP-2 and MMP-9), promoting tumor metastasis and PMN development in prostate cancer [96].

### Platelets promote PMN development in bone metastasis

**Fig. 2:**
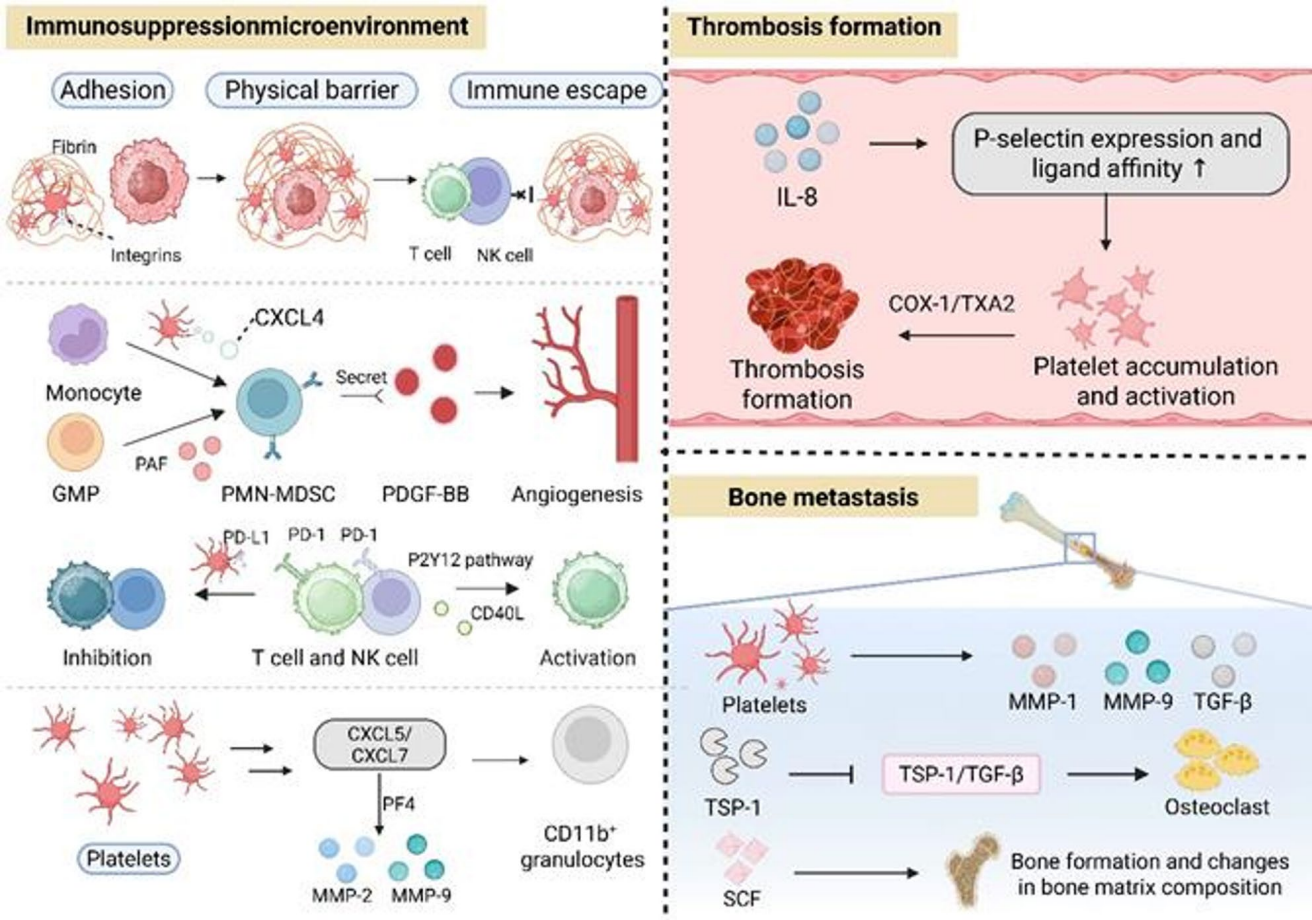
The multiple roles of platelets in accelerating the formation and development of PMN. Platelets play a pivotal role in PMN establishment by fostering both a thrombotic and an immunosuppressive microenvironment. As key mediators of hemostasis, they promote tumor cell-endothelial adhesion and thrombus formation, which drives metastasis and PMN maturation. Furthermore, platelets act as dual modulators of immune evasion: they form physical barriers that shield tumor cells from surveillance and engage in direct crosstalk with immune cells (particularly MDSCs and T cells). Finally, platelets synergize with tumor-secreted bone metabolism-related molecules to facilitate osteotropic colonization, thereby reinforcing the metastatic cascade in skeletalsites

Bone is a common cancer metastatic site, with cure extremely challenging once metastasis occurs. Notably, patients often show abnormal bone resorption/formation prior to bone metastasis [97]; these abnormalities intensify with cancer progression, driving bone metastasis. Primary tumor cells regulate bone physiology to facilitate pre-metastatic bone microenvironment colonization. Platelets mediate primary tumor-bone crosstalk, absorbing tumor-secreted bone metabolism-related molecules (TGF-β, MMP-1/9) to regulate bone microenvironment remodeling and bone PMN formation, promoting bone metastasis [98, 99]. In prostate cancer, platelet-derived SCF induces bone formation and matrix component changes [100]; platelet thrombospondin-1 (TSP-1, a classic anti-angiogenic protein) is markedly elevated in tumor-bearing mice. Via the TSP-1/TGF-β axis, TSP-1 inhibits osteoclastogenesis and regulates bone PMN formation, enhancing prostate cancer bone metastasis [101]. platelets critically contribute to PMN formation via protective coating, thrombosis, immune modulation, and ECM remodeling (Fig. 2).

Current studies confirm platelets drive PMN formation via tumor cell activation, thrombosis, immunosuppression, and bone-specific modulation, but key unresolved gaps limit full understanding and translation: Existing research focuses on individual platelet pathways but lacks clarity on hierarchical/synergistic activation networks and molecular switches for PMN maturation, obscuring how platelets precisely orchestrate PMN formation [102]. Downstream signaling between platelet-derived molecules (CXCL4, PDGF-BB) and immune cell recruitment/reprogramming (MDSCs, T/NK suppression) is uncharacterized, leaving unclear how platelets form the immunosuppressive PMN protecting CTCs [103]. Organ-specific platelet dependence (platelet-independent liver vs. dependent lung/bone PMNs) highlights poor understanding of how platelets integrate with organ microenvironments and regulators (TDSFs, BMDCs) to shape tissue-specific niches, critical to explaining metastatic organotropism [104]. These gaps hinder translation: lacking PMN-specific biomarkers for platelet activity, early pre-metastatic detection is elusive, and separating platelet-mediated PMN regulation from normal hemostasis blocks therapies targeting metastasis without disrupting vascular function. Closing these gaps is key to moving beyond descriptive insights to targeted PMN inhibition.

## Fibrinogen mediates PMN establishment

Fibrinogen, a coagulation factor derived from hepatocytes, is a large soluble glycoprotein in serum that polymerizes into fibrin when thrombin - activated, acting as a key acute phase protein in coagulation to prevent bleeding in pathology [105]. Our previous review has established that fibrinogen is also an essential TME mediator, promoting PMN establishment and growth by enhancing immunosuppression, inflammation, angiogenesis, and ECM remodeling [5]. In solid tumor patients, elevated serum fibrinogen stems from VEGF-driven VP and autocrine secretion by tumor cells like MCF-7 (which deposit fibrinogen into the TME) [106]. High levels of primary tumor-derived fibrinogen can further migrate to metastatic sites, reprogramming host stromal cells to shape the PMN via inflammatory and pro-angiogenic microenvironments.

### Fibrinogen elicits inflammatory PMN and the post-translational modifications of fibrinogen

Fibrinogen is key inflammatory and prognostic indicator for tumor metastasis and plays a key role in regulating the formation of an inflammatory PMN [5]. Immune cells express multiple fibrinogen-binding receptors, and these receptor-ligand interactions can either activate inflammatory signaling pathways or induce the secretion of various pro-inflammatory cytokines (e.g., IL-6 and TNF-α) [107]. Specifically, fibrinogen enhances and amplifies inflammatory responses through various signaling pathways, such as the IL-8-CXCR2 and extracellular regulated kinase (ERK) pathways [108, 109]. The interactions of fibrinogen and macrophages induces the expression of macrophage-associated chemokines such as MIP-1α, MIP-1β, MIP-2, and MCP-1 [110]. Fibrinogen binds to the αMβ2 integrin on macrophages, thereby activating FAK and suppressing the p53/14-3-3σ pathway to promote the proliferation and growth of CRC cells [111]; this interaction is also intimately associated with diverse inflammatory responses through the activation of the nuclear factor-κB (NF-κB) signaling pathway [112].

Fibrinogen’s role in the TME has gained growing attention recently. Abnormal post-translational modifications (PTMs: citrullination, phosphorylation, proteolysis) are closely linked to tumor inflammation, promoting PMN development and metastasis. Fibrinogen citrullination correlates with higher inflammatory protein levels [113]. Pancreatic cancer patients have markedly elevated plasma hydroxyfibrinogen [114, 115], while ovarian cancer patients show increased phosphorylated fibrinogen [116]. Additionally, prolyl 4-hydroxylated α-fibrinogen contributes to αFG-565HyP production (a molecule strongly linked to cancer/inflammation) [114]. These findings suggest that PTMs of fibrinogen can enhance inflammation. Future research should focus on elucidating the precise mechanisms by which these modifications influence the TME, particularly their effects on VP and their interplay with immune cells to foster immunosuppression and angiogenesis. A deeper understanding of these processes is crucial for uncovering fibrinogen’s role in PMN formation and identifying potential therapeutic targets.

### Fibronogen promotes an immunosuppressive PMN

Fibrinogen interacts with diverse immune cells (T/B/NK cells, macrophages, MDSCs), driving immunosuppressive TME, tumor evasion, metastasis, and PMN formation [5]. It adheres to tumor cells, forming a fibrin matrix that shields them from NK cell cytotoxicity, promoting tumor growth/metastasis/evasion [117]. MDSCs are key for TME immunosuppression [118]; Han et al.. showed tumor-derived TF-induced fibrin deposition increases CD11b + Gr-1 + MDSC recruitment in lung cancer [119]. Takada et al.. first reported fibrinogen γC399tr binds T cell integrin αMβ2 to mitigate autoimmunity [120]. Fibrinogen also interacts with macrophages, stimulating MIP-1α/β, MIP-2 secretion to modulate T cell function [110], and regulates macrophage cytokine/chemokine release, promoting accumulation/M2 polarization and altering immune function. Additionally, fibrinogen induces ICAM1 to recruit macrophages to gallbladder cancer liver metastasis [121].

The fibrinogen-like protein (FGL) family (especially FGL1/FGL2) is critical for cancer immune evasion [122]. FGL1 is the primary ligand for T cell LAG3 [123], and the FGL1/LAG3 axis drives tumor EMT, immune escape, and immune checkpoint blockade resistance [124]. FGL1-LAG3 binding induces functional exhaustion of CD8⁺ tissue - resident memory (TRM) cells, characterized by reduced secretion of cytotoxic molecules (e.g., granzyme B, IFN - γ) and upregulated expression of inhibitory receptors (e.g., LAG3, PD − 1), rather than a decrease in cell numbers [125, 126]. This binding facilitates liver metastasis. Besides its role in liver metastasis, FGL1 also has other functions. In CRC liver metastasis, elevated FGL1 reduces IFN - γ + CD8+/CD4 + and Ki67 + CD8+/CD4 + T cell infiltration via TAM - OTUD1 - FGL axis, thereby promoting immunosuppressive liver TME [127]. In addition to the effects of FGL2 on CD8 + T cells, it also plays a role in gliomas. FGL2 binds CD8 + T cell FcγRIIB to induce apoptosis and limit immune responses [128]; FGL2 in glioblastoma inhibits brain tumor CD103 + DC differentiation and suppresses CD8 + T cells via NF-κB/STAT1/5/p38 pathways [125]. Soluble FGL2 attenuates DC- mediated CD8 + T/Th1 cell activity, inducing liver cancer immunosuppression [126]. In gliomas, FGL2 binds macrophage CD16 to release CXCL7 and promote recruitment [129]. Recent studies show FGL2 regulates MDSC differentiation/immunosuppressive function via XBP1 axis, modulating CRC MDSC cholesterol biosynthesis [29].

### Fibrinogen promotes angiogenesis in the PMN

Fibrinogen and its degradation fragments have potent pro-angiogenic activity, with distinct structural domains exerting different angiogenic effects. Our recent work identifies fibrinogen as key for PMN vascularization via VE-cadherin downregulation and endothelial junction disruption, favoring tumor colonization [14]. Fibrinogen directly interacts with VEGF/FGF [130], enhancing their levels and endothelial migration in PMN to promote angiogenesis, tumor growth, and metastasis. Mechanistically, fibrinogen-growth factor binding prevents proteolytic degradation and maximizes endothelial cell presentation. For example, fibrinogen co-administration with VEGF/bFGF has an additive effect, enhancing growth factor activity [131]. Notably, fibrinogen-specific fragment-endothelial interactions clarify its molecular angiogenic mechanism: FGA’s RGD site (572–574) binds activated endothelial integrin αvβ3 [132], sending signals to promote adhesion, migration, and angiogenesis. fibrinogen promotes endothelial proliferation/angiogenesis independently of growth factors. Sahni et al. showed fibrinogen stimulates HuDMEC proliferation/migration with or without bFGF/VEGF165 [130]. Unlike growth factors, fibrinogen enhances vessel number/coverage [133], confirming a growth factor-independent angiogenic pathway.

Fibrinogen accumulates in tumor ECM during tumor metastasis/PMN formation, providing a temporary matrix for new vessels [134]. fibrinogen is converted to fibrin, forming a matrix that scaffolds endothelial migration/proliferation, critical for neovascularization [135]. However, certain fibrinogen domains hinder secondary organ PMN formation. For instance, fibrinogen γ chain’s COOH-terminal globular domain (γC) induces endothelial apoptosis, inhibiting CRC angiogenesis/metastasis [136]. Similarly, Dejana et al.. showed fibrinogen E fragment (fibrinogenE) inhibits endothelial migration [137].Notably, FGL family (FGL1/FGL2) plays versatile roles in angiogenesis/PMN formation [138]. Bie et al.. showed FGL1 ablation stimulates proliferation, EMT, and angiogenesis in LKB1-mutant lung adenocarcinoma [139]; Okan et al.. showed LKB1 suppresses angiogenesis via Rab7-mediated neuropilin-1 degradation [140]. FGL2 promotes HCC tumor growth/angiogenesis in a thrombin-dependent manner [141]. FGL2 silencing in HCCLM6 cells (hFGL2(low) HCCLM6) retards xenograft growth and angiogenesis, potentially linked to reduced ERK/JNK phosphorylation [141].

### Fibrinogen drives ECM remodeling and EMT regulation

ECM provides structural support and reshapes the TME to favor metastasis [142]. Fibrinogen acts as an ECM scaffold to facilitate cell adhesion, modulate cytokine activity, and stimulate cellular responses, as exemplified by its interaction with leukocyte β2 integrins and melanoma ICAM-1 [143]. It also synergizes with fibronectin to boost brain tumor-initiating cell (BTIC) invasiveness and metastatic potential [144]. Critical to ECM remodeling is fibrinogen’s regulation of MMPs: it modulates MMP-2/9 activity to degrade ECM components, creating migration routes for tumor cells [144]. Fibrinogen further recruits TAMs into the TME; recruited TAMs secrete MMP-3/9 to reprogram ECM structure, and stimulate CAFs via reactive oxygen species (ROS) to activate matrix metalloenzymes, amplifying ECM remodeling and PMN shaping [145, 146]. Additionally, primary tumor macrophages reprogram ECM through protease activity and collagen interactions, forming channels for metastable cancer cells [147].

EMT is essential for tumor metastasis, with E-cadherin, N-cadherin, and vimentin as key markers [148]. Fibrinogen overexpression upregulates the mesenchymal marker N-cadherin while downregulating the epithelial marker E-cadherin, inducing an invasive mesenchymal phenotype [149]. In clear cell renal cell carcinoma, FGL1 exerts similar effects by reducing E-cadherin and increasing N-cadherin [150]. Conversely, FGA inhibits gastric cancer cell proliferation, motility, and EMT by suppressing ITGA5 and blocking the FAK/ERK pathway [151]. Fibrinogen amplifies EMT via TAM crosstalk: TAM-derived TNF-α and TGF-β activate and stabilize Snail through the NF-κB/β-catenin pathway, forming an EMT positive feedback loop [146].

**Fig. 3:**
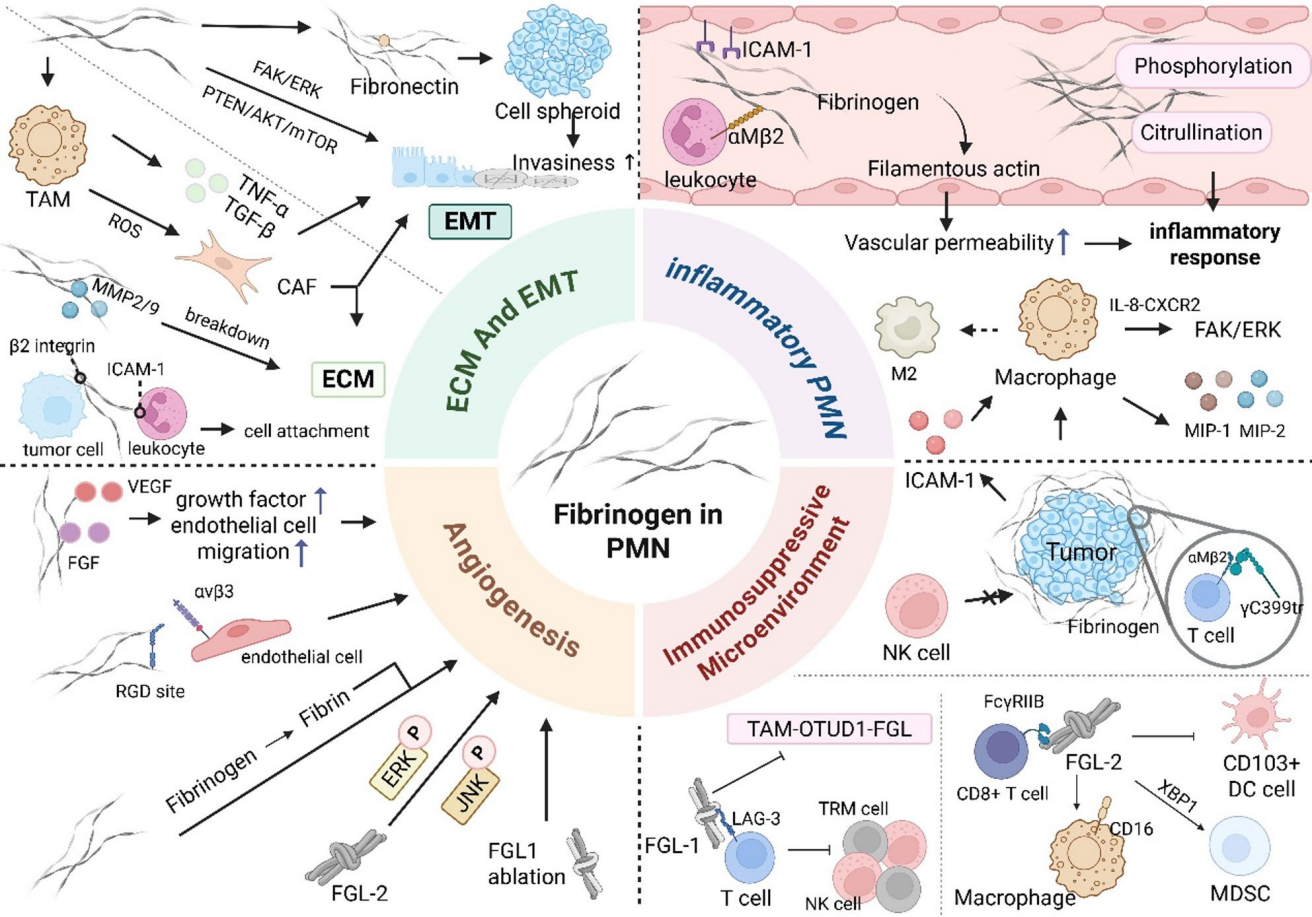
Fibrinogen facilitates the establishment of PMN. Fibrinogen orchestrates PMN establishment and tumor metastasis by coordinating inflammation, immunosuppression, angiogenesis, ECM remodeling, and EMT. First, it drives inflammation by enhancing vascular permeability and promoting the release of inflammatory cytokines, with aberrant PTMs further amplifying this pro-inflammatory state. Second, through extensive crosstalk with immune cells—including recruitment of MDSCs, modulation of T-cell dysfunction, and regulation of macrophage polarization—fibrinogen fosters an immunosuppressive microenvironment, a process reinforced by the FGL family via pathways such as FGL1/LAG3 and XBP1. Third, in angiogenesis, fibrinogen interacts directly with growth factors (e.g., VEGF, FGF) to increase their local concentrations in the PMN and accelerate endothelial cell migration, thereby promoting neovascularization. Finally, fibrinogen promotes EMT by altering E-cadherin/N-cadherin/vimentin dynamics and facilitates ECM remodeling through MMP activation and disruption of intercellular adhesion. Collectively, these processes synergize to prime metastatic sites and enable efficient tumor cell dissemination

Above in all, fibrinogen primarily enhances PMN formation by fostering immune suppression, driving inflammation, angiogenesis, and ECM remodeling (Fig. 3) . [5, 130, 152]. Clarifying these mechanisms will advance our understanding of metastasis and aid in the development of effective targeted treatments [5].

## The role of thrombin and tissue factor in PMN formation

In a typical coagulation process, TF serves as the primary physiological initiator, triggering the coagulation cascade to generate thrombin [153]. TF and thrombin play pivotal roles in shaping a pro-metastatic microenvironment within the tumor milieu: they promote fibrin deposition, enhance platelet activation, and activate signaling pathways such as protease-activated receptors (PARs), which mediate downstream cellular responses, thereby accelerating tumor growth, angiogenesis, and distant metastasis [154]. Notably, thrombin overexpression is closely associated with cancer progression, including tumor growth, PMN formation and metastasis, as it facilitates fibrin formation, activates platelets, and induces thrombosis [10].

### Thrombin drives PMN establishment

Thrombin orchestrates TME remodeling and PMN establishment via three major, distinct molecular pathways. First, it regulates ECM remodeling at metastatic organs by inducing the release of matrix-degrading enzymes (e.g., MMPs): thrombin-PAR1 signaling upregulates MMP-2/9 in nasopharyngeal carcinoma to enhance invasion [155], and MMP-2/13 in chondrosarcoma (with PAR1/4 involvement) to promote migration [156]; it also activates platelets to secrete α-granule MMPs for further matrix degradation [157]. Second, thrombin promotes angiogenesis and vasculogenic mimicry (VM) through VEGF-dependent and -independent mechanisms: it increases VEGF secretion in multiple cell types [158].drives VM via PAR-1/NF-κB [159], and upregulates VEGF via HIF-1α/p44/42 MAPK in gliomas [160]. Third, it shapes an immunosuppressive microenvironment: in pancreatic ductal adenocarcinoma, the thrombin-PAR1 cascade upregulates Csf2 and Ptgs2 to induce immunosuppressive gene expression and accelerate tumor progression [161].

Thrombin exerts its pro-metastatic function by activating platelets via protease-activated receptors (PARs), notably PAR1 and PAR4, and this activation event simultaneously promotes the secretion of angiogenic factors (e.g., VEGF), thereby contributing to tumor metastasis [10]. Despite this well-characterized role in tumor progression, the specific functions of this classical thrombin-PAR signaling pathway during PMN formation has not yet been defined. Further studies are required to validate it.

### TF regulates PMN formation

TF drives PMN formation through three interconnected mechanisms: ECM remodeling, angiogenesis, and immune cell crosstalk. For ECM remodeling, TF upregulates matrix-degrading enzymes including MMPs and urokinase-type plasminogen activator (uPA). In breast cancer, the TF/FVIIa complex induces MMP-2 expression via the PI3K/AKT-NF-κB pathway, thereby enhancing matrix degradation and ECM remodeling [162]. Independent of MMPs, TF also mediates matrix degradation through uPA: Del Rosso et al. demonstrated that TF/FVIIa upregulates uPAR in pancreatic cancer, which in turn drives ECM degradation via the uPA/uPAR axis [163]. In terms of angiogenesis and tumor-endothelial interaction, TF signaling promotes tumor-endothelial attachment through endothelial α3/β1 integrins; for example, breast cancer cells engage with β1 integrins on human umbilical vein endothelial cells (HUVECs) to enhance adherence and subsequent metastasis [164]. Elevated FVII levels in breast cancer correlate with disease progression and liver metastasis, a process linked to enhanced EMT and cell invasion; notably, tumor-derived FVII promotes metastasis, whereas liver-derived FVII exerts an inhibitory effect [165]. TF-driven angiogenesis is further associated with VEGF over-expression: coagulation-dependent activation of TF/VIIa triggers MAPK signaling—a key regulator of VEGF expression [166]. Moreover, PAR-dependent crosstalk between tumor cells and host vascular cells facilitates metastasis [167]; TF upregulation stimulates thrombin production, which enhances signaling via PARs on tumor and stromal cells, ultimately promoting metastasis through the TF-thrombin-PAR1 axis [168]. Soluble alternatively spliced TF (flTF)/FVIIa-mediated PAR2 activation also induces the secretion of pro-angiogenic factors (VEGF, IL-8, CXCL1) to fuel angiogenesis [169]. Finally, TF contributes to immune cell recruitment for PMN formation by attracting CD11b⁺ cells, which implies a potential role in the recruitment of BMDCs to the PMN [170].

## Drugs targeting coagulation factors to inhibit tumor metastasis

Targeting coagulation factors represents a promising yet clinically challenging anti-metastatic strategy: its core mechanism involves disrupting PMN formation to inhibit tumor metastasis, as exemplified by the use of anti-platelet agents or anticoagulants [171]. However, this approach is hindered by two key limitations: an inherent risk of bleeding and the difficulty of achieving tumor-specific targeting [172, 173]. Future breakthroughs are likely to stem from three complementary directions: the development of selective coagulation factor inhibitors, the stratification and targeted treatment of hypercoagulable patient populations, and the combination of coagulation-targeted therapies with existing anti-cancer modalities. Ultimately, the clinical translation of this strategy will require rigorous benefit-risk assessments and the implementation of innovative delivery or targeting strategies.

### The benifits of anti-coagulation approaches in patients

#### Platelet therapeutic targets

Platelets are pivotal drivers of tumor progression, regulating polymorphonuclear myeloid-derived suppressor cell formation, immunosuppressive TME establishment, and thrombosis to facilitate tumor immune escape and distant metastasis. Large-sample clinical studies confirm that advanced melanoma patients receiving combined immunotherapy and anticoagulants exhibit significantly improved survival, underscoring the therapeutic potential of targeting platelet-related pathways [174]. Antiplatelet agents show promising anti-metastatic effects (Table 1): aspirin reduces incidence and metastasis of liver, gastric, head and neck, and ovarian cancers [175–179]; P2Y12 antagonists (e.g., clopidogrel) inhibit ADP-dependent GPIIb/IIIa activation to block platelet-tumor cell crosstalk [180–182]; GPVI antagonist JAQ1 suppresses platelet activation, reduces breast cancer cell aggregation and extravasation in vitro, and alleviates lung metastasis in mouse models, while its Fab2 fragment induces intratumoral hemorrhage to boost chemotherapeutic efficacy [183–186]. Novel therapeutic strategies and targets have emerged from platelet research: platelet-derived growth factor B (PDGFB) expression correlates with clinical outcomes, with low levels improving lung cancer survival [187]. Bioengineering approaches include a platelet-tPA system that disperses CTCs clusters [188], and PLGA particles coated with platelet membranes for targeted drug delivery in metastatic breast cancer [189]. Moreover, regulating platelet-related cell interactions—such as blocking PAF/TAP-mediated crosstalk or the CXCL4 pathway—alters MDSC differentiation to suppress metastasis [87–89], while PD-1/PD-L1 blockade mitigates platelet-T cell crosstalk and restores anti-tumor immunity [90–92].

**Table 1:** Clinical use of various coagulation factors in PMN

Therapeutic Modality & Target	Compound name	Mechanism	References
Platelets targeted nano drugs	Biomimetic Anisotropic-Functionalized Platelet-Membrane-Coated Polymeric Particles	Through codelivery of TRAIL and doxorubicin, the particles’ tailored attachment to metastatic human breast cancer cells resulted in increased cell death	[189]
Fibrinogen targeted therapy drugs	Anticoagulants (AC) (heparin, warfarin) and fibrinolytic agents (FA) (urokinase, protease)	Counteracts excessive clotting around tumor tissue and inhibits cancer metastasis	[190]
TF targeted ADC	TF-011-MMAE (monomethyl auristatin E)	Interruption of TF: FVII-dependent intracellular signaling, cytotoxic MMAE-mediated tumor killing	[203]
	SC1-MMAE	Inhibition of the TF-PAR2-dependent MAPK/ERK phosphorylation cascade and cytotoxic MMAE-mediated tumor killing	[227]
	SC1-DM1(emtansine)	Inhibition of TF-PAR2-dependent MAPK/ERK phosphorylation cascade	[227]
	Tisotumab vedotin	Upon endocytosis MMAE disrupts microscopic polymerisation and mediates tumor killing	[228]
TF ligand inhibitor	Rivaroxaban	Inhibition of FX activation targets PAR2 signaling for reprogramming of TAM	[229]
	PCI-27,483	Inhibits TF: FVIIa complex and PAR2 downstream signaling	[230]
TF pathway inhibitor	Recombinant nematode anticoagulant protein c2 (rNAPc2)	Inhibits the formation of TF-VIIa complexes with antithrombotic properties and inhibits the initiation of complex enzyme linkages in the coagulation process	[231]
	Warfarin (loanword)	Blockade of TF/VIIa complex and inhibition of TF-mediated angiogenesis	[166, 169]
P2Y12 receptor antagonists	Clopidogrel	Blocking ADP-stimulated platelet activation and inhibiting platelet aggregation	[232]
	ticagrelor	Blocking ADP-stimulated platelet activation and inhibiting platelet aggregation	[178]
GPVI receptor antagonists	JAQ1	Blockade of GPVI, the predominant platelet activation receptor, inhibits platelet activation	[183]
	Revacept	Blockade of GPVI, the predominant platelet activation receptor, inhibits platelet activation	[178]
PDPN mAbs	NZ-1	Targeting PDPN protein on platelets to inhibit PDPN-mediated platelet aggregation and cancer metastasis	[233]
	MS-1	Targeting PDPN protein on platelets to inhibit PDPN-mediated platelet aggregation and cancer metastasis	[234]
Thrombin inhibitor	Hirudin	Increased ERK1/2 inactivation status and down-regulation of typical MAPK/ERK signaling pathway expression	[212]
	DTIP	Inhibit the formation of VM and tumor metastasis	[159]
Thrombin receptor inhibitor	Vorapaxar	Inhibition of thrombin-activated receptor PAR1 and its downstream pathway	[216]
	Imidazopyridazine compound I-191	Inhibition of thrombin-activated receptor PAR2 and its downstream pathway	[235]

#### Fibrinogen therapeutic targets

Agents for tumor-associated coagulation disorders mainly include anticoagulants (ACs: warfarin, heparin [190–193]) and fibrinolytic agents (FAs: urokinase, protease [190, 194]). They exert anti-tumor effects by inhibiting fibrin formation, reducing hypercoagulability and metastasis risk [190]. Costantini et al. confirmed ACs suppress metastasis of small-cell lung cancer, renal cancer, and melanoma, while FAs act on NSCLC, breast, colon, and prostate cancers [190]. However, these agents have limitations: narrow applicability as adjuvant therapies, and excessive/prolonged use disrupts normal coagulation, elevating bleeding risk and requiring continuous monitoring that burdens patients [195, 196]. Beyond coagulation, fibrinogen drives tumor progression via regulating immunosuppression, angiogenesis, and ECM remodeling, offering novel therapeutic targets. The FGL1/LAG3 axis mediates tumor EMT and immune evasion; its inhibition may reduce metastasis [124, 197, 198]. In NSCLC, blocking the KDM4A-STAT3 pathway suppresses FGL1 transcription and tumor progression [199]. In glioblastoma, FGL2 impairs dendritic cell function via the NF-κB/STAT1/5/p38 pathway, implying its inhibition may prevent PMN formation [125].

#### TF and thrombin therapeutic targets

TF is highly expressed in metastasis-prone organs and the TME to drive PMN development; alternatively spliced TF (asTF) also recruits TAMs and boosts pro-inflammatory cytokine secretion (TNF-α, IL-6) [200, 201]. Corresponding therapies include the neutralizing antibody RabMab1 (blocks asTF to inhibit pancreatic cancer PMN [202]), antibody-drug conjugate TF-011-MMAE (suppresses TDSFs in solid tumors [203]), and vitamin K antagonist warfarin (reduces prostate cancer incidence and sensitizes pancreatic cancer to chemotherapy [204, 205]) (Table 1). TF/FVIIa blockade additionally inhibits breast cancer MMP-2 via the PI3K/AKT-NF-κB pathway [162], while near-infrared photoimmunotherapy (NIR-PIT) improves survival in cancer models [206].

Thrombin, another critical factor, is targeted by agents like hirudin, which shows efficacy across tumor types [207–212]—vincristine-conjugated recombinant hirudin inhibits melanoma growth and metastasis [213], and bifunctional hMnSOD-hirudin fusion proteins suppress lung cancer invasion [214]. PAR1 inhibitors (vorapaxar, atopaxar) regulate ECM remodeling and angiogenesis, though clinical validation is limited [155, 215–219]. Mechanistically, thrombin promotes VM via PAR-1-NF-κB signaling, which novel inhibitors (R-Hirudin, DTIP) can block [159]. Additional advances include recombinant haemathrin 2 (inhibits thrombin-induced cell migration [220]), dabigatran derivatives (enhance sorafenib efficacy in HCC [221]), and fibrinogen-thrombin systems (disrupt tumor metabolic flux [222]).

### The risks of anti-coagulation approaches in patients

Despite the promising anti-metastatic potential of antiplatelet and anticoagulant therapies outlined above, these interventions are accompanied by notable risks and limitations that cannot be overlooked. First and foremost, bleeding complications represent the most direct and prevalent risk: antiplatelet agents such as aspirin and clopidogrel inhibit platelet aggregation, while anticoagulants suppress the coagulation cascade, and although these drugs act through different mechanisms, both ultimately impair the body’s hemostatic ability. This elevates the risk of spontaneous bleeding—ranging from mild events such as gingival bleeding and bruising to severe, life-threatening conditions like intracranial hemorrhage, gastrointestinal bleeding, and hemoptysis. For cancer patients, the risk is further amplified: malignancy itself often induces a hypercoagulable state, and invasive procedures commonly used in cancer treatment (e.g., surgery, chemotherapy, radiotherapy) can damage blood vessels, exacerbating bleeding tendencies.

In addition to bleeding risks, the therapies are plagued by issues related to drug efficacy and patient tolerability. On one hand, their anti-metastatic effects are not universal across all cancers—for instance, while aspirin shows benefits in gastrointestinal, head and neck, and ovarian cancers, its efficacy in other tumor types (e.g., pancreatic cancer, glioma) remains unclear or inconsistent in clinical studies [223]; long-term use may also lead to acquired drug resistance, such as reduced platelet responsiveness to P2Y12 antagonists, which diminishes therapeutic effects over time [224]. On the other hand, off-target effects and adverse reactions compromise tolerability: aspirin can irritate the gastrointestinal mucosa, leading to ulcers, abdominal pain, and dyspepsia even in low-dose regimens; highly specific GPVI antagonists like JAQ1 may disrupt normal platelet functions (e.g., wound healing, pathogen defense) beyond tumor-related targets, increasing infection risk and delaying tissue repair. Notably, the intratumoral hemorrhage induced by JAQ1’s Fab2 fragment—though beneficial for chemotherapeutic accumulation—could cause unexpected intratumoral bleeding in some patients, especially those with advanced, highly vascularized tumors [186].

Thirdly, Complex drug-drug interactions pose significant hurdles to clinical management, a challenge that is particularly pronounced in cancer patients, who typically receive multi-drug regimens encompassing chemotherapy, targeted therapy, and immunotherapy [225]. For instance, antiplatelet and anticoagulant drugs may interact adversely with these cancer-directed agents: combining antiplatelet drugs with angiogenesis inhibitors can heighten bleeding risks, while co-administration with immunotherapy may disrupt immune cell function in unpredictable ways. Critically, these interactions have the potential to alter treatment efficacy, and clear clinical guidelines to address them are currently lacking [226].

## Perspectives and conclusions

**Fig. 4:**
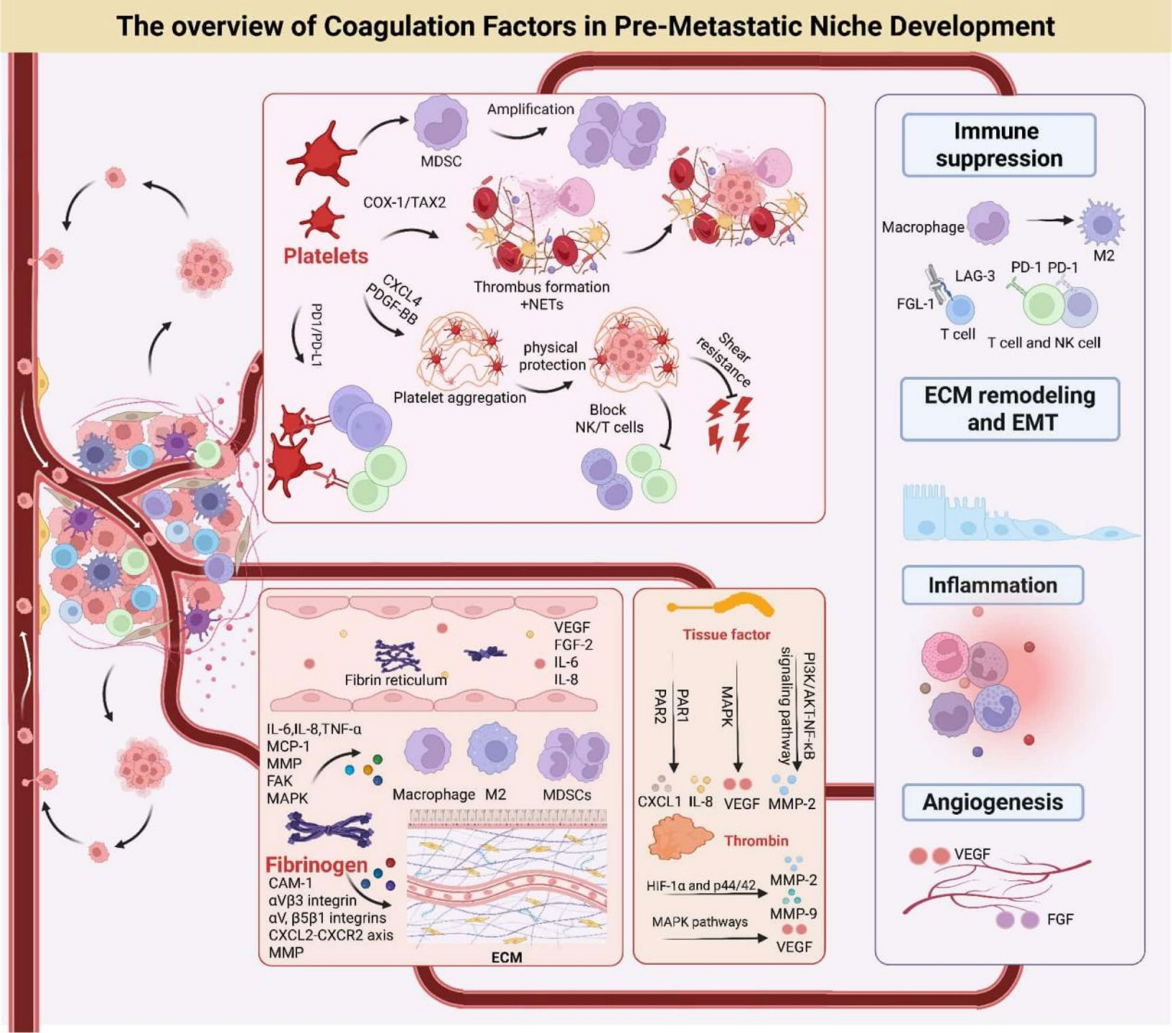
The overview of coagulation factors in PMN Development. Coagulation factors, including platelets, fibrinogen, tissue factors, and thrombin, are crucial for establishing the PMN. Platelets can activate endothelial cells, recruit immune cells, and release pro-angiogenic factors to facilitate tumor cell colonization and growth. Fibrinogen interacts with immune cells, promotes inflammation, angiogenesis, and ECM remodeling, creating a favorable microenvironment for CTCs. Thrombin and TF are closely associated with ECM remodeling and angiogenesis in PMN, primarily mediating ECM remodeling via MMP and driving angiogenesis and tumor metastasis through PAR. Collectively, these factors interact with tumor cells and the stroma to establish an immunosuppressive, inflammatory, angiogenic and ECM-remodeled PMN , ultimately facilitating metastatic colonization

Cancer metastasis is the leading cause of cancer-related deaths. Preventing metastasis is a major challenge in cancer therapy. The formation of the PMN is a critical step in the metastatic process. PMN is a favorable microenvironment for the colonization and growth of CTCs in distant organs. Coagulation factors, traditionally known for their role in hemostasis and thrombosis, have recently been implicated in PMN formation and metastasis. This review summarizes the current understanding of the role of coagulation factors in PMN formation and metastasis (Table 2). We discuss how coagulation factors, such as platelets, fibrinogen, and thrombin, can promote PMN formation through various mechanisms (Fig. 4). Platelets can activate endothelial cells, recruit immune cells, and release pro-angiogenic factors to facilitate tumor cell colonization and growth. Fibrinogen interacts with immune cells, promotes inflammation, angiogenesis, and ECM remodeling, creating a favorable microenvironment for CTCs. Thrombin and TF are closely associated with ECM remodeling and angiogenesis in PMN, primarily mediating ECM remodeling via MMP and driving angiogenesis and tumor metastasis through PAR.

**Table 2:** Roles of various coagulation factors in the formation of the PMN

Coagulation Factor Type	Core Function	Key Mechanism	Key Signaling Pathways / Molecules	References
PLT	Drive PMN formation, accelerate tumor metastasis	Activated by tumor cells (direct binding, soluble factors, EV transmission)	PSGL-1/P-selectin, PD-L1/PD-1, CXCL4, PDGF-BB, MMP-2/9, SCF, TSP-1	[71–73, 90, 91, 96, 101, 105, 236]
		Promotes PMN thrombus formation		
		Constructs an immunosuppressive microenvironment		
		Regulates bone metastasis PMN development		
Fibrinogen	Remodel PMN microenvironment, support CTC colonization	Induces inflammatory response and abnormal post-translational modifications	IL-8-CXCR2, NF-κB, FGL1/LAG3, VEGF/FGF, αvβ3, MMP-2/9, FAK/ERK	[110, 114, 115, 122, 123, 125, 149, 237]
		Regulates immune cell interactions, constructing an immunosuppressive PMN		
		Promotes angiogenesis		
		Drives ECM remodeling and EMT		
Thrombin	Regulate PMN function, strengthen metastatic microenvironment	Induces the release of matrix-degrading enzymes, promoting ECM remodeling	PAR1/PAR4, MMP-2/9/13, VEGF, HIF-1α/p44/42 MAPK, Csf2, Ptgs2	[155, 157, 238, 239]
		Drives angiogenesis and angiogenic mimicry		
		Activates the expression of immunosuppressive genes		
TF	Initiate and coordinate PMN formation, mediate multi-faceted regulation	Upregulates matrix-degrading enzymes, promoting ECM remodeling	PI3K/AKT-NF-κB, uPA/uPAR, VEGF, MAPK, PAR2, CD11b⁺ BMDCs	[158, 159, 162, 166]
		Enhances tumor-endothelial adhesion, driving angiogenesis		
		Recruits CD11b⁺ immune cells		

Coagulation factors are critical for PMN formation and metastasis, which suggests that targeting these factors could represent a promising anti-metastatic therapeutic strategy. Nevertheless, two key challenges must be addressed before such strategies can be translated into clinical practice: first, the risks and benefits of these approaches need to be carefully balanced for individual patients; second, our current understanding of how coagulation factors specifically influence PMN formation remains limited. Many questions remain unclear and need to be further explored: (1) PMN formation occurs in a specific spatio-temporal sequence, yet the roles of coagulation factors at each stage and the pathways involved remain unclear. (2) In the PMN formation process driven by various coagulation factors, additional receptors and signaling pathways require further investigation. (3) EVs plays a crucial role in promoting PMN formation and tumor metastasis. Further investigation is needed to understand how various clotting components and EVs interact to facilitate tumor progression. (4) Current research investigates the pathway of PMN promotion by individual coagulation factors. Whether interactions among multiple coagulation factors work synergistically or antagonistically to enhance PMN formation deserve further researches. (5) While multiple coagulation factors serve as important biomarkers for assessing tumorigenesis and metastasis, their precise sensitivity and specificity for diagnosing and predicting tumor prognosis necessitate further investigation through large-scale, multicenter studies. (6) The benefits and drawbacks of anticoagulant therapy for suppressing metastatic niche function must be thoroughly weighed. By addressing these research directions, we can gain a better understanding of the role of coagulation factors in metastasis and develop novel therapeutic strategies to prevent this deadly process.

## Data Availability

Not applicable.

## Abbreviations

CTCs: Circulating tumor cells
TME: Tumor microenvironment
PMN: Pre-metastatic niche
TDSFs: Tumor-derived soluble factors
BMDCs: Bone marrow-derived cells
ECM: Extracellular matrix
VEGF: Vascular endothelial growth factor
CRC: Colorectal cancer
MDSCs: Myeloid-derived suppressor cells
Tregs: Regulatory T cels
CAFs: Cancer-associated fibroblasts
MMP: Matrix metalloproteinase
TAMs: Tumor-associated macrophages
HCC: Hepatocellular carcinoma
TXA2: Thromboxane A2
PF4: Platelet factor 4
TSP-1: Thrombospondin-1
VP: Vascular permeability
ICAM-1: Intercellular adhesion molecule-1
ERK: Extracellular regulated kinase
FAK: Focal adhesion kinase
PTMs: Post-translational modifications
FGL: Fibrinogen-like protein
LAG3: Lymphocyte activation gene 3
FGF: Fibroblast growth factor
EMT: Epithelial-mesenchymal transition
PARs: Protease-activated receptors
VM: Vasculogenic mimicry
TF: Tissue factor
